# Exploiting Information in Event-Related Brain Potentials from Average Temporal Waveform, Time–Frequency Representation, and Phase Dynamics

**DOI:** 10.3390/bioengineering10091054

**Published:** 2023-09-07

**Authors:** Guang Ouyang, Changsong Zhou

**Affiliations:** 1 Faculty of Education, The University of Hong Kong, Hong Kong; 2 Department of Physics, Centre for Nonlinear Studies, The Beijing-Hong Kong-Singapore Joint Centre for Nonlinear and Complex Systems (Hong Kong), Institute of Computational and Theoretical Studies, Hong Kong Baptist University, Kowloon Tong, Hong Kong

**Keywords:** EEG, ERP, time-frequency analysis, machine learning, phase dynamics, single trials

## Abstract

Characterizing the brain’s dynamic pattern of response to an input in electroencephalography (EEG) is not a trivial task due to the entanglement of the complex spontaneous brain activity. In this context, the brain’s response can be defined as (1) the additional neural activity components generated after the input or (2) the changes in the ongoing spontaneous activities induced by the input. Moreover, the response can be manifested in multiple features. Three commonly studied examples of features are (1) transient temporal waveform, (2) time–frequency representation, and (3) phase dynamics. The most extensively used method of average event-related potentials (ERPs) captures the first one, while the latter two and other more complex features are attracting increasing attention. However, there has not been much work providing a systematic illustration and guidance for how to effectively exploit multifaceted features in neural cognitive research. Based on a visual oddball ERPs dataset with 200 participants, this work demonstrates how the information from the above-mentioned features are complementary to each other and how they can be integrated based on stereotypical neural-network-based machine learning approaches to better exploit neural dynamic information in basic and applied cognitive research.

## 1. Introduction

To understand how the brain processes external information by evoking a series of neural cognitive activities, a key approach is to characterize the patterns of brain response signals and identify neural variables from the patterns that are associated with the manipulations of the cognitive processes. This approach has been dominating the research in cognitive neuroscience irrespective of what recording technology is used. Through this approach, we can obtain a progressively improved understanding of the meaning of various neural activity patterns in terms of what functional or cognitive processes they represent, thereby developing tools to better exploit the brain’s signals for life enhancement, such as the brain–computer interface (BCI).

However, there still exist substantial research gaps regarding how the brain’s response can be better characterized given the large amount of concurrent noise [1,2,3,4]. The major obstacles in characterizing the brain’s response can be summarized as follows. (1) The brain’s activity signals, both the intrinsic and response-related ones, are mixed with intrinsic noise and measurement noise [1,3]. (2) The brain’s response is highly variable and susceptible to variations in external factors [5,6]. (3) The brain’s response can be manifested in a variety of features that cannot be feasibly characterized by a single method or algorithm [7,8,9]. (4) There are multiple forms of response that are mixed with each other, including the ones that are evoked by external stimulus and the ones that comprise the change of ongoing spontaneous activities [10,11]. Each one of them may be composed of multiple sub-components. Given the complexity, as summarized above, recent advances in brain signal analysis suggest a composite and multifaceted approach be used to characterize the data in order to understand the fairly complex cognitive processes, which can further advance the applied domain of neural cognitive sciences [9]. For instance, in brain–computer interface research, thoroughly identifying all aspects of neural activity change related to a cognitive process of interest (e.g., mental representation of a language symbol) is the key approach used to achieve a high information transfer rate and thus high practicality [12,13]. Due to the issues mentioned above, using a single feature characterization method is far from being sufficient. Our present work will focus on the relevant issues based on electroencephalography (EEG) and its associated brains response characterization methods, such as average event-related potentials (ERPs) and the event-related spectral perturbation method (ERSP). The brain’s response pattern characterized from EEG signals inherits all the issues described above. For example, a stimulus input usually leads to evoked ERP components (e.g., P1-N1 complex) and the suppression of alpha power due to general task engagement [14,15,16,17], and these response activity components are embedded in strong noises. The two response patterns also overlap in the frequency range. However, the commonly used average ERPs method is only able to reveal the former because the alpha power suppression cannot be shown in the average waveform due to phase stochasticity. Likewise, the time–frequency power (or amplitude) analysis is able to reveal changes in the non-phase-locked oscillation power, but would not be able to reveal the phase-resetting (without amplitude change) of ongoing oscillations [18]. The measurement of phase synchronization features across trials (e.g., phase-locking index) complements ERPs and time–frequency power analysis in revealing the dynamics of phase information across different frequencies [19]. Because all of these different ways of characterizing the brain’s response capture information that is distinct from each other, it is imperative to integrate multiple methods in order identify or describe the neural activity features associated with a cognitive process.

Although it is conceptually clear that different facets of information from complex biological signals like neural activity need to be integrated in both basic and applied neuroscience research, there have not been systematic guidelines established or a demonstration created regarding how to implement it. The objective of our current work is therefore to present a detailed and complete set of procedures that are able to integrate multifaceted neural features characterized by different methods and demonstrate their benefits in neural cognitive research, particularly in examining the relationship between neural data and cognitive factors (cognitive manipulations). Following this objective, we will select three frequently examined aspects of features in EEG-based brain response signals, systematically present how a cognitive effect is manifested in different aspects of features in a non-redundant way, and examine how to integrate the different aspects of features to better reveal the cognitive effect on the neural dynamics. The data analysis routines can be extended to more than three features and other neural data modalities. The selected three aspects of the features are as follows: (1) The average ERP waveform (will be referred to as ERP later). This feature is undoubtedly the most used feature in EEG-based neural cognitive research. The ERPs method is a simple average of the segments (frequently called epochs) of EEG time-locked to a specific event (e.g., stimulus presentation). The ERPs method is effective in removing noise but sacrifices non-phase-locked brain response activity. (2) Oscillation power in the time–frequency space (will be referred to as the TF power later). The TF power is able to show the brain’s response throughout the time–frequency space in both the enhancement and suppression of the oscillation power at different frequency bands compared to the baseline level (before the event), irrespective of whether the phases of the oscillations are synchronized to an event. (3) Phase dynamics of the oscillations. Since the power of the oscillations in the time–frequency space does not reveal the phase information, theoretically, it is expected that the phase information is not redundant information related to the TF power, but encodes additional information about the neural cognitive processes or effects.

To summarize, it is a key endeavor to identify more comprehensive neural dynamics changes associated with an external factor of interest (e.g., sensory, perceptual, cognitive, mental imagery, motor) in the broad field of cognitive and applied neuroscience research. In the present work, we will use an EEG dataset with 200 participants performing a typical visual oddball task for demonstration. In these data, the cognitive factor of interest is the brain’s processing of different degrees of unexpectedness, i.e., the neural dynamic activity generated by the rare stimulus should be different from that generated by the frequent stimulus. And such neural dynamics changes should be multifaceted and integrated to better reveal the effect of unexpectedness processing.

## 2. Method and Results

This tutorial-like paper will combine the Method section and Results section for a better presentation flow. The structure of the present section is as follows: (1) Description of the EEG dataset used and its associated cognitive task. The task is a simple oddball task that represents a generic paradigm of a serial stimulus presentation in which there are discrete time points eliciting the brain’s responses. (2) Description of three different ways to calculate the brain’s response (event-related potential (ERP), time–frequency (TF) power, and phase dynamics) and how these three different methods show different patterns. (3) Demonstration of the non-redundancy of cognitive information encoded in different neural features based on machine learning. The demonstration is based on trial-average features (i.e., the average of the brain’s response pattern from the same condition (or type) of trial). (4) Same as (3), but demonstration of the non-redundancy based on a single-trial brain response.

### 2.1. EEG Dataset Used

The EEG dataset used for demonstration was obtained from a classic visual oddball task with 200 participants [20]. A total of 200 participants (62 males, 138 females, 18–40 years old, mean: 25.1, SD: 4.5) performed a visual oddball task. In the task, the participants watched a sequence of 160 color squares (135 blue, 24 red, 1 yellow) presented one-by-one on the screen with a duration of 200 ms. The task was to count how many different colors there were in the sequence (the answer is three and was unbeknown to the participants). The blue and red were counterbalanced across participants, i.e., for half of the participants, it was 24 blue, 135 red, 1 yellow. Only blue and red squares were used here, serving as frequent and rare conditions, respectively. The inter-stimulus interval (ISI) was uniformly distributed between 1700 ms and 2700 ms. EEG data were collected in a sound-attenuated room using Brain Product’s actiCHamp amplifier with 32 channels. The reference channel was the amplifier’s GND electrode connected to the mid-point between Fp1 and Fp2. The following pre-processing steps were conducted on the data: (1) down-sampling to 150 Hz; (2) bandpass filtering between 1 and 45 Hz; (3) applying the SPA method [21] as a coarse-grained procedure to remove large amplitude artifacts; (4) applying extended Infomax ICA algorithm in the EEGLAB toolbox [22,23] to decompose the data; and (5) applying ICLabel to remove ocular artifacts with a probability larger than 0.5 [24]. The scripts for this study are available online at https://github.com/guangouyang/STF accessed on 5 September 2023.

### 2.2. Calculation and Presentation of the ERP, TF Power, and Phase Dynamics

The EEG data were epoched at the window of [−1000 ms, +2000 ms] with respect to the stimulus onset. The ERPs were directly averaged from the epochs separately for different conditions. Since rare and frequent conditions have different numbers of trials (24 v.s. 135), a subset (24 trials) of the frequent trials was randomly drawn for statistical analysis (see details below), but the full set was used for visualization.

For the TF power and phase dynamics analysis, we applied wavelet transform based on the following procedure. First, the wavelet transform was applied on each single-trial based on the standard formula:

$$W\left(a,b\right)=a^{-1/2}\int_{-\infty }^{+\infty }S\left(t\right)\psi^{*}((t-b)/a)dt$$

Here, *W* is the coefficient function, a is the scaling parameter (the inverse of a is linearly linked to frequency), *b* is the translational parameter (representing different time points), *S*(*t*) is the signal (here, a single epoch of EEG), *ψ*(*t*) is the wavelet function, and * denotes the complex conjugate. In the present application, we chose the complex morlet wavelet with a time–decay parameter of 0.5 as the wavelet. The time–decay parameter value was chosen to balance the time and frequency resolution in the current data. The coefficient in every time–frequency point *W* is a complex value whose modulus and angle serve as an estimate of the amplitude and phase, respectively. Note that the TF power here was directly defined and calculated as the amplitude (not the square of it).

**Figure 1:**
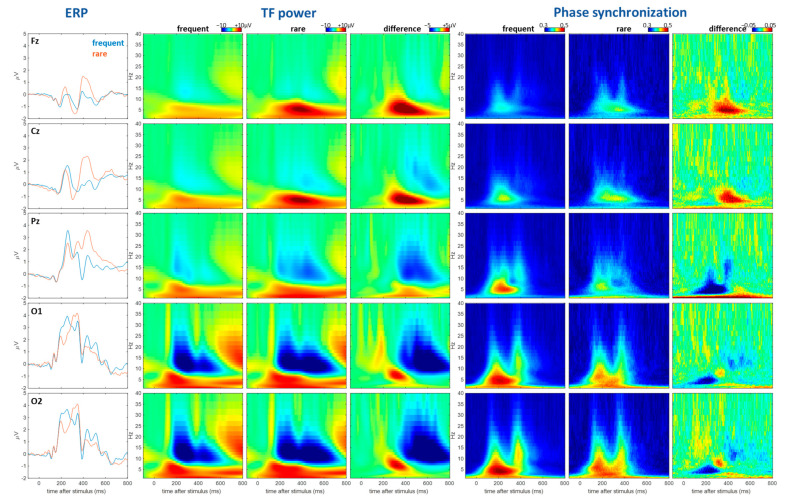
Brain’s response pattern characterized by different methods. Frist column: the ERP waveforms from different electrodes (red: rare condition; blue: frequent condition). Second to fourth column: the time–frequency (TF) power. Different rows are from different electrodes, as described in the first column. The second and third columns are from rare and frequent conditions. The fourth column is the difference between rare and frequent conditions. Fifth to seventh columns: same organization as the second to fourth columns but for phase synchronization data.

For phase dynamics, we mainly calculated the synchronization of phases across single trials following the procedures below: at each time point and frequency, the phases for all trials were collected and equally divided into 10 bins. The cumulated probability of each bin was calculated by dividing the count in each bin by the total counts. Each probability is denoted by *p_i_* (*i* from 1 to 10). Finally, the standard deviation of the probability across the 10 bins, $\sqrt{\sum {\left(p_{i}-\mu \right)}^{2}/10}$ (where *µ* is the mean), characterizes the synchronization across trials. To explain, if the phases are not synchronized at all, i.e., they are randomly distributed, the probability in each bin will be equal, thus leading to the low standard deviation of the counts across bins, and vice versa. This index is similar to the phase-locking value/index [25], but since the calculation here is much simpler, we simply termed it “phase synchronization”. All response patterns calculated here are baselined to the [−200 ms, 0 ms] window. The ERPs, TF power, and phase synchronization for five representative electrodes are presented in Figure 1.

Figure 1 shows the descriptive features of the brain’s response characterized by different methods, i.e., the ERP, TF power and phase synchronization. The main descriptive points are summarized as follows: (1) The conditional effect (difference between rare and frequent) is visually distinguishable across all characteristics. For the ERP, it is manifested as the difference in the transient temporal waveform, mainly comprising the early visual mismatch negativity [26] and novelty-related P3 [27]. For the TF power (Figure 1, 2–4th columns), both the enhancement (red) and suppression (blue) of the oscillation power are shown in the brain response activity, and these patterns have clear time and frequency locations. Such rich information regarding the brain’s response patterns is not available in ERPs. In addition, both the enhancement (more in central frontal region) and suppression (more in occipital regions) of the oscillatory activity show clear condition effects (Figure 1, 4th column). Finally, the phase synchronization plots (Figure 1, 5–7th columns) again show substantially different patterns compared to the TF power, demonstrating that the phase aspects provide non-redundant information regarding cognitive effects. Only an increase in the phase synchronization can be found in the response patterns (5–6th columns), which is theoretically expected because the pre-stimulus activity should have minimal phase synchronization (i.e., zero). The minimal value of phase synchronization shown in Figure 1 is around 0.3, which is caused by the intrinsic fluctuation in the data. It is noteworthy that both an increase and decrease in the phase synchronization are found in the rare condition compared to the frequent one (see the difference pattern in the 7th column), and this difference pattern is distinct from the difference pattern in the TF power (4th column); this indicates, again, non-redundant information associated with cognitive effects. It is important to note here that a decrease in the phase synchronization does not necessarily mean a weaker response component that is overwhelmed by noise; it could mean a larger trial-to-trial variability without a diminishing amplitude. For instance, the TF power at Pz does not show clear a reduction in the amplitude in the region of 100–300 ms and 3–7 Hz (Figure 1, 4th column, Pz); however, the phase synchronization in the region was greatly reduced (Figure 1, 7th column, Pz), implying a predominant increase in the trial-to-trial variability.

The conditional difference shown in the ERP (Figure 1, 1st column), TF power (4th column), and phase synchronization (7th column) are all highly significant. However, we will not present the conventional statistical analyses (e.g., *t* tests, non-parametric tests) here for two reasons: (1) the conventional analyses do not provide novel insights here and are not within the scope of the present work; (2) the machine-learning-based approach, as will be shown later, can better show the generalizability of the conditional effect as it is based on a separate training and testing dataset scheme. The main scope of the present work is to demonstrate the non-redundancy of different features and how to integrate them to better capture conditional effects (thus, cognitive effects). Below, we will continue to analytically present the non-redundancy of different features.

In sum, from the patterns in Figure 1, we can identify three major types of brain responses:

1. R1: Transient dynamic responses that are additive to the ongoing activity.
2. R2: Suppression of ongoing oscillatory activity.
3. R3: Enhancement of ongoing oscillatory activity.

R2 and R3 are non-phase locked and, apparently, are not present in the ERPs. R2 and R3 are mainly manifested as the suppression and rebound of the alpha band oscillation that are clearly present in the TF power but not in the phase synchronization plot. Note that if a response mainly belongs to the R1 type, there will be high consistency in the patterns of the TF power and phase synchronization plot. For example, the theta band response occurring at around 200–600 ms at the central and frontal region (Cz, Fz) clearly belongs to R1, as shown by the consistent pattern between the TF power and phase synchronization plot.

### 2.3. Demonstration of the Non-Redundancy of Cognitive Information Encoded in Different Neural Features Based on Machine Learning (on Trial-Average Features)

To re-iterate the main rationales of the present work, the brain response patterns characterized in different ways should encode different aspects of the information related to a specific cognitive process of interest in a way that shows that they are complementary to each other. Therefore, it is desirable to integrate different aspects of neural features when studying the association between neural dynamics and cognitive processes. In this section, we apply a machine learning approach to demonstrate the statements above.

**Figure 2:**
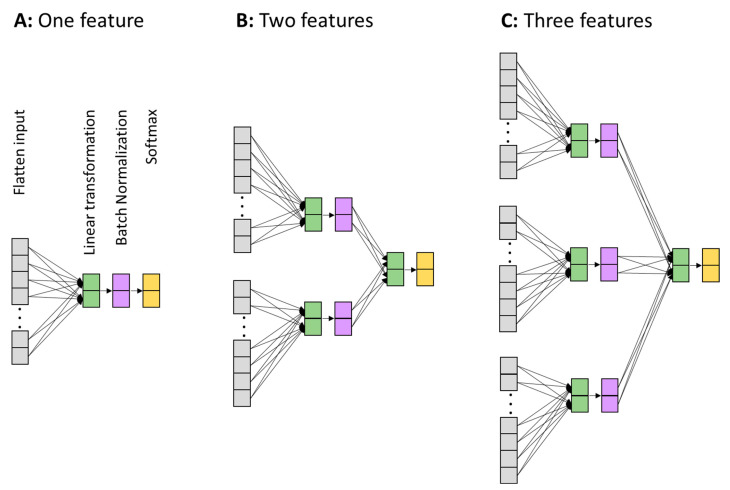
Architectures of neural network classifiers. (**A**) Network for intaking one feature (ERP or TF power or phase information). (**B**) Network for intaking two features (any combination of two). (**C**) Network for intaking the three features together. Gray: vectorization layer—turns the input data into a 1-D vector. Green: linear transformation layer. Purple: batch normalization layer. The batch normalization layer is mainly introduced to make the output from different features (**B**,**C**) at a similar scale before feeding into the final linear transformation layer. Orange: SoftMax layer. The output of the last layer, the SoftMax layer decides which condition the network predicts: rare or frequent.

Classifier model. We used a generic perceptron neural network to learn and classify the data samples into rare and frequent conditions (labeled as 1 and 0, respectively). The neural networks are depicted in Figure 2. Three networks were built, each one intaking a different number of data features (1, or 2, or 3). The three features were the ERP, TF power, or phase information. For the one-feature case (Figure 2A), the network received and flattened the data sample from all time points, electrodes, and frequencies (for TF and phase) into a 1-D vector. The flattened layer was fully connected to the next linear transform layer with two elements corresponding to the two conditions in the current application. The linear layer’s two-value output was batch-normalized in order to be comparable with the composite network (Figure 2B,C), which required homogenizing the output values of different features. Finally, the softmax layer was applied to make decisions and calculate the decision error.

Machine learning procedures and parameters. The machine learning application was first applied to the individual participant’s average brain response patterns. For the ERP, two types of data samples were generated: the average ERP from the rare condition and the average ERP from the frequent condition. To ensure that the trial number did not confound the machine learning classification, both were averaged from 24 trials (maximum trials available for rare condition). Therefore, there were 5 average ERPs for the frequent condition (each one from 24 randomly drawn non-overlapping trials of the frequent condition) and 1 average ERP for the rare condition for each participant. For each single iteration of the machine learning full procedure, only one (randomly drawn) frequent sample was taken, thus creating a dataset of 400 samples (200 participants with rare and frequent ERPs). For these 400 samples, 100 samples were randomly drawn to serve as the test data and the other 300 samples served as the training data. For each iteration, this 100–300 split was re-performed. In sum, the machine learning approach based on the one-feature network (Figure 2A) was trained on the 300 samples and tested on the unexposed 100 samples to classify them into rare and frequent conditions. The validation accuracy from the test data was reported; this was calculated by the number of correctly identified samples (*n_c_*) divided by the number of total samples (*n_all_*) in the validation sample, i.e., *n_c_/n_all_*. The sample data arrangement was also applied to the TF power and phase information. When the application was applied to the average responses, the phase information used was the phase synchronization metric (see its calculation above). The optimizer used was the stochastic gradient descent (SGD) with a learning rate of 1 × 10^−4^ and a momentum of 0.9. The parameter of momentum uses the changes in the past to counter the non-essential stochastic fluctuations in the gradient descent updates, similar to the concept of acceleration in physics [28]. The value ranges from 0 to 1, with 0 meaning no momentum at all and 1 meaning a full consideration of the previous update. The error function used was the cross-entropy loss between the predicted logits and actual labels: $L_{CE}=-\sum p\left(x\right)\log q\left(x\right)$. Here, *p* and *q* are the probability functions of the actual label and predicted logits. For the average data samples, the batch size was 50 and the training and testing were run for 200 epochs. The entire machine learning procedure (with 200 epochs) was run 200 times. In each iteration, the randomization for the k-fold and selection of the frequent data sample were re-drawn. The statistical results from the 200 iterations of the entire procedure are reported.

The machine learning procedures described above were the same when applied to each of the three individual features (ERP, TF power, phase synchronization), but not for the composite features. For the composite features (Figure 2B,C), the initial weights for the linear transformation layer were directly taken from the learned weights from the individual trained networks (Figure 2A). This procedure is understandable because it is heuristically much more guided and closer to the final solution for the composite models to start with the trained states, compared to starting with totally random ones. In addition, more complex models would be more likely to sink into local minima if starting from randomness. Therefore, starting the composite models in a way that is guided by the trained simple models is considered to be beneficial.

**Figure 3:**
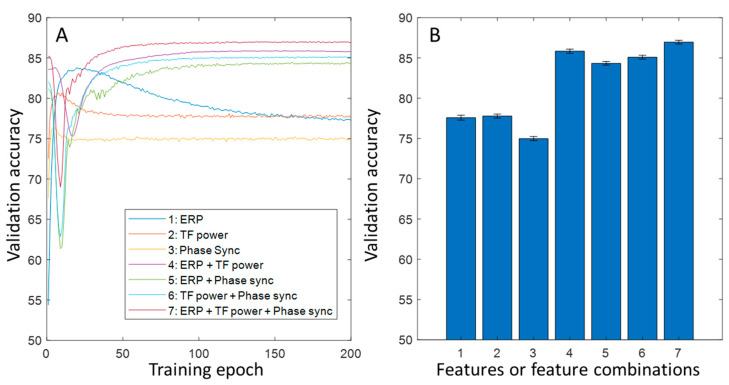
Validation accuracy of classifying rare and frequent conditions on the average ERPs from different models. (**A**) Development of validation accuracy over training epochs. (**B**) Error bars showing means and standard error of means for different models using different features of a combination of features (the indices are noted in (**A**)).

The development of the validation accuracy across the 200 epochs averaged from the 200 realizations is shown in Figure 3. As expected, the composite models all converged to higher validation accuracies (Figure 3A), with the three-feature model reaching the highest accuracy (86.9%).

### 2.4. Demonstration of the Non-Redundancy of Cognitive Information Encoded in Different Neural Features Based on Machine Learning (on Single-Trial Features)

Based on the procedure described above, we further explored the data at single-trial levels. First, we expected that the basic hypothesis, i.e., combining more aspects of neural features will extract more cognitive information, would hold true at the single-trial level. Therefore, the results shown in Figure 3 should retain their overall pattern in the single-trial data. Second, because the single-trial EEG data are much noisier compared to the trial-average version, we expected the validation accuracy to be significantly lower.

**Figure 4:**
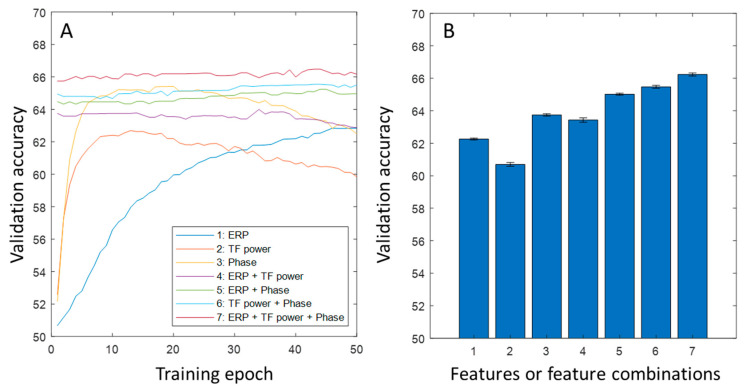
Validation accuracy of classifying rare and frequent conditions on the single-trial ERPs from different models. (**A**) Development of validation accuracy over training epochs. (**B**) Error bars showing means and standard error or means for different models using different features of a combination of features (the indices are noted in (**A**)).

Since each participant had 24 rare trials, we randomly selected 24 frequent trials for the machine learning application. After pooling all participants together, there were 24 × 200 = 4800 samples labeled as “rare”, and 4800 samples labeled as “frequent”. The batch size changed from 50 to 1920. Due to the computational cost, the epoch was reduced to 50. In the single-trial data, phase synchronization did not exist (as it is a cross-trial concept). Therefore, simple phase information from the time–frequency wavelet parameter was used. The development of the validation accuracy across the training epochs and the bar plots comparing the results from the last 20 epochs are shown in Figure 4.

## 3. Discussion

### 3.1. Summary

The present work presented the average brain response patterns and their association with cognitive factors, characterized using different methods, namely the time course of event-related potentials, the time–frequency power, and phase dynamics. It was first descriptively shown that different characterization methods display different brain response information patterns (Figure 1), and it was later demonstrated, using the machine learning approach, that different features encode different information in a way that means they are complementary to each other, leading to the conclusion that a combination of different features is able to better exploit neural information for research or applications in cognitive science.

### 3.2. Implications for Basic Neural Cognitive Research

In conventional EEG-based cognitive research, many researchers follow a prototypical procedure for studying brain–cognition–behavior relationships, that is, identifying a classic “component” and studying how this component covaries relative to the experimental manipulation of a cognitive process. One famous example is the N400 component, which is believed to encode semantic processing and has a clear set of features in terms of the time course and scalp topography [29]. We propose that it is worth re-thinking the meaning of an ERP component defined as such and exploring a more comprehensive way of characterizing the neural substrate of a cognitive effect. It is conceptually clear that the average ERP waveform is a partial (if not distorted) representation of the neural dynamic response because it cancels out the non-phase-locked oscillatory components that also respond to stimulus input (clearly shown in Figure 1). In a similar vein, phase information, including the single-trial value and cross-trial feature (e.g., synchronization), also clearly codes the brain’s response in a unique way (Figure 1). Therefore, researchers may consider applying data analysis approaches that can integrate different aspects of features and examine the brain–cognition–behavior relationships through this approach. One powerful and popular approach is the machine learning framework, which is also demonstrated in the present article. Take N400 as an example, one can examine the validation accuracy of classifying brain responses in semantic violated and non-violated conditions based on the combination of multiple features, and further study how the validation accuracy is affected by any other factor of interest. Another advantage of using machine learning approaches is that they have a higher generalization power. In machine learning analysis, the training and testing data are not communicated in any way. This is different from traditional statistical analyses that are applied to the whole dataset, and is thus more susceptible to the issue of circular reasoning because the parameters (e.g., time window, electrodes) are commonly heuristically determined by the gross feature [30].

### 3.3. Implications for Applied Neuroscience Research

In applied neuroscience research, such as that on the brain–computer interface, exploiting as much relevant information from the collected neural data as possible is one of the main goals. The current results clearly demonstrated that the integration of multiple features led to the better exploitation of cognitively relevant information. Following the success of generative pre-trained transformers (GPTs), the machine learning models trained from existing data can serve as a decoder for future classifications or real time decoding. Although the present application demonstrated that the integration of multiple features improves the classification accuracy, the accuracy did not reach a high value. One of the reasons is that 200 participants, although relatively large for a typical basic cognitive study, is far from enough for training a high-performance neural decoder. One potential improvement is to integrate more features. In the present application, we used three features to demonstrate the basic principle, but this can be extended to more (or much more) than three features. For example, we can include features of cross-frequency coupling, scale-free dynamics, microstates, entropy, and so on. However, a potential pitfall of including many features is that the classifier might more easily sink into the local minima if the training data are not sufficient or if the network architecture is not appropriate. In general, involving more features requires a proportionally improved network model and an increased data size.

### 3.4. The Accuracies at Different Levels of Machine Learning Applications: Single Trial and Average Data

In our machine learning applications, the validation accuracy reached 86.9% for the composite features, which means that the classifier made a mistake for every 7.6 samples of the average brain response patterns from any individual participant. This accuracy is not high in a generic sense. However, considering the commonly recognized large cross-individual variability in brain activity patterns [31], it is promising that the machine learning approach, when combined with multiple neural features, could lead to a potentially powerful non-invasive data decoder in the future. This accuracy value drops to 66.4% at single-trial level, which is significantly lower. The most likely reason for this drop would be the substantially higher noise in the single-trial data. While the upper limit of the validation accuracy in the current application is unknown due to the unknown amount of intrinsic noise (some noise could be signaled but not yet detected due to the limitation of current algorithms), there are several ways to improve the accuracy in such applications. The first one is to add new features (see examples mentioned above) to the set of training data. In principle, the more independent the new features are from the existing features, the more the accuracy will be improved. The second one is to develop more sophisticated neural network architectures. The current architectures are basically linear models that do not exploit high-level features, particularly the internal relationships between different low-level features. One typical example of internal relationships is the spatial pattern (inter-electrode relationships), which is supposed to be better exploited by convolutional neural network (CNN)-type networks. The other example of internal relationships is the time series pattern, which is supposed to be better exploited by recurrent neural network (RNN)-type networks. Finally, the transformer-type neural network would also possibly lead to much more powerful exploitation; however, this is only on the basis of a sufficient amount of training data. The scope of the current work is only to use the machine learning approach to demonstrate the non-redundancy of information coded in different features, and not to demonstrate a high accuracy level in any sense.

### 3.5. Comparison with Conventional Approaches to Analyzing Neural Activity Data

In a typical paradigm of neural cognitive research, researchers usually characterize the descriptive patterns of the associations between neural signals and cognitive factors first and then apply statistical testing to the associations in a way that has been informed by the found association to a certain degree [30]. These conventional approaches have two intrinsic limitations. The first one is the circularity issue, as mentioned above [30]. The second one is that the descriptive effects (or between-condition differences) may not be able to reveal those effects that are invisible to researchers but are visible to machine-learning-based classifiers. Moreover, applying machine learning approaches based on the test–validation data split approach can automatically address the issue of circularity. The key challenge that remains in the machine learning approach is the requirement of a large sample size for training. This issue may be addressed by incorporating a pre-trained model approach in the future.

### 3.6. Limitations

The main limitations of the present demonstration lie in the following two aspects. The first one is that only simple linear and low-order information was exploited. This means that the machine learning approach mainly exploited the simple magnitude difference in the neural features (ERP, TF power, phase synchronization) between the two different conditions. In principle, higher-order and nonlinear features can be further exploited to reveal more detailed and complex relationships between neural signals and cognitive variables. This can be implemented in two ways. The first method is to directly apply signal processing to extract the complex and nonlinear features before feeding them to the neural networks. The other method is to directly apply complex neural network architectures that are able to capture complex features. The second limitation is that we did not exploit the intrinsic patterns in the multivariate data, i.e., the spatiotemporal patterns formed by the internal relationships between the data time points, electrodes, frequencies. One example that exploits such information is EEG microstates [7,32]. Likewise, exploiting such intrinsic patterns should be based on neural architectures that are equipped with such capacities. These complex neural network architectures (CNN, RNN, transformers [31,33,34]) could be used combinatorically to achieve such purposes, which, however, would require a much larger number of data samples.

### 3.7. Future Directions

The current work demonstrates the non-redundancy of several key features in the EEG data associated with specific cognitive processes. The results support the potential to better exploit neural dynamic information based on multifaceted feature characterization methods combined with machine learning approaches. Based on the rationales summarized above, three directions that may bear significant research value in exploiting neural information are proposed here: (1) the development of more multifaceted, theoretically informed, non-overlapping feature characterization methods, particularly methods that exploit the internal spatial–temporal structures; (2) the development or application of sophisticated neural network architectures to the multi-faceted neural data; (3) the collection, assembling or generation of large-sized data to utilize the full potential of complex neural networks. While collecting data may be costly, assembling and generating data could be a higher-priority option. Assembling here refers to combining multiple datasets from different sources. Generating refers to developing specific application settings that can obtain more data samples from existing datasets. For example, for time series analysis and prediction, every time point in the EEG data stream can serve as a single data sample, thus creating a much higher sample size compared to epoch-based applications.

## Data Availability

The datasets and code for the current study are available from the corresponding author.
